# Changes on risky drinking after the COVID-19 outbreak in Brazil: results from three consecutive web surveys

**DOI:** 10.47626/2237-6089-2023-0686

**Published:** 2025-05-26

**Authors:** Luisa Alencar Santos Lage, Fátima Smith Erthal, Marcelo Ribeiro-Alves, Aline Furtado Bastos, Vicent Balanzá-Martinez, Flavio Kapczinski, Raquel B. De Boni

**Affiliations:** 1 Universidade Federal do Rio de Janeiro Instituto de Psiquiatria Rio de Janeiro RJ Brazil Instituto de Psiquiatria (IPUB), Universidade Federal do Rio de Janeiro (UFRJ), Rio de Janeiro, RJ, Brazil.; 2 UFRJ Instituto de Biofísica Carlos Chagas Filho Rio de Janeiro RJ Brazil Instituto de Biofísica Carlos Chagas Filho (IBCCF), UFRJ, Rio de Janeiro, RJ, Brazil.; 3 Fundação Oswaldo Cruz Instituto Nacional de Infectologia Evandro Chagas Rio de Janeiro RJ Brazil Instituto Nacional de Infectologia Evandro Chagas (INI), Fundação Oswaldo Cruz (FIOCRUZ), Rio de Janeiro, RJ, Brazil.; 4 University of Valencia Department of Medicine Teaching Unit of Psychiatry and Psychological Medicine Valencia Spain Teaching Unit of Psychiatry and Psychological Medicine, Department of Medicine, University of Valencia, Network Centre for Biomedical Research in Mental Health CIBERSAM, Valencia, Spain.; 5 Universidade Federal do Rio Grande do Sul Departamento de Psiquiatria Porto Alegre RS Brazil Departamento de Psiquiatria, Universidade Federal do Rio Grande do Sul (UFRGS), Porto Alegre, RS, Brazil.; 6 Hospital de Clínicas de Porto Alegre Porto Alegre RS Brazil Hospital de Clínicas de Porto Alegre (HCPA), Porto Alegre, RS, Brazil.; 7 FIOCRUZ Instituto de Comunicação e Informação Cientifica e Tecnológica em Saúde Rio de Janeiro RJ Brazil Instituto de Comunicação e Informação Cientifica e Tecnológica em Saúde (ICICT), FIOCRUZ, Rio de Janeiro, RJ, Brazil.

**Keywords:** Alcohol, chronic diseases, mental health disorders, developing countries, surveys, internet

## Abstract

**Objective:**

Risky drinking (RD) is associated with an increased risk of chronic and infectious diseases, injuries, and violence. This study aimed to assess changes in RD in Brazil after coronavirus disease 2019 (COVID-19) outbreak, both overall and among individuals with self-reported chronic diseases and mental health disorders.

**Methods:**

We conducted three independent, anonymous web surveys in Brazil including adult participants: Survey 1 (S1) (April/2020, n = 19,257), S2 (August/2020, n = 1,590), and S3 (January/2021, n = 859). Participants were recruited through adapted snowball sampling and sponsored social network advertisements. RD was assessed using the Alcohol Use Disorder Identification Test-Concise (AUDIT-C), designed to identify individuals at risk of alcohol-related problems. Logistic regression analyses with bootstrapping (B = 2,000) were performed, with stratification by sex, age, education, employment, household size, and the presence of chronic and mental health conditions, as well as lifestyle factors, to address sample imbalances.

**Results:**

The estimated prevalence of RD was 45.8% (95% confidence interval [95%CI] 45.5-46.1) in S1, 35.3% (95%CI 34.9-35.6) in S2, and 33.7% (95%CI 33.3-34.0) in S3. Participants with chronic diseases consistently presented lower RD prevalence across all three surveys, compared to those without such conditions. Conversely, individuals with mental health disorders presented higher RD prevalence than those without such diagnoses in S1 and S2, but not in S3.

**Conclusion:**

Despite the decrease in RD prevalence, monitoring of alcohol consumption trends remains essential for shaping effective public health policies. Additionally, the observed variations among individuals reporting chronic and mental health disorders highlight the need for targeted interventions in future crises.

## Introduction

The harmful use of alcohol is a public health problem and a risk factor for morbidity and mortality worldwide.^1-3^ For these reasons, its decrease is one of the United Nations’ sustainable development goals.^4^ At the beginning of the coronavirus disease 2019 (COVID-19) pandemic, there was huge concern that the crisis could lead to an increase on alcohol consumption.^5^ In 2022, a systematic review evaluated the changes on alcohol consumption during the pandemic and found heterogeneous results. Studies showed significant decreases in total alcohol consumption and binge drinking, and significant increase in the frequency of alcohol use and alcohol use disorder (AUD) in some countries, but not in others.^6^ Furthermore, many studies indicated that the majority of individuals kept their drinking at the same levels as before the pandemic.^7-12^

In Brazil, cross-sectional studies presented unconclusive results regarding alcohol consumption during the COVID-19 pandemic either reporting its reduction^13,14^ or increase.^15^ However, none of those studies was longitudinal and only Nin et al.^16^ investigated alcohol consumption through a validated scale (Alcohol, Smoking and Substance Involvement Screening Test [ASSIST]). The mixed results may be explained by different methodologies, and by the substantial risk of recall bias (since the changes on alcohol consumption relied on self-reports from retrospective behavior).

As per the last US Preventive Services Task Force, "‘Risky’ or hazardous alcohol use means drinking more than the recommended daily, weekly, or per-occasion amounts, resulting in increased risk for health consequences but not meeting criteria for alcohol use disorders (AUD)."^17^ During the beginning of the COVID-19 pandemic, we found the prevalence of risky drinking (RD) at 45.5%.^18^ It is noteworthy that the prevalence of AUD/RD may be higher in clinical populations when compared to the general population. For example, in some Brazilian studies, the prevalence of an AUD positive screening was around 13% in patients seen in primary care, while severe AUD ranged from 9.8% among patients in primary care, and 12 and 14% among individuals with positive screening for depression and anxiety, respectively.^19-23^ On the hand, Salles et al.^24^ did not find statistically significant differences in AUD prevalence among individuals reporting chronic diseases (7.5% [95% confidence interval {95%CI 6.1-8.7}]) and mental health disorders (8.4% [95%CI 6.7-10.2]). Considering the burden of the pandemic over the health system, evaluating the use of alcohol among individuals with comorbidities brings important information for planning contingency strategies in health. Moreover, investigating the pattern of alcohol consumption during a pandemic may orient the development of prevention strategies to face future challenges like the COVID-19 pandemic. Despite the great relevance of this topic, few studies were conducted in Latin America during the COVID-19 pandemic. The largest was conducted in Colombia and Mexico and found a decrease on alcohol use among patients at primary health services,^25^ but authors acknowledge that this may reflect a decrease in the number of patients seeking for the such services.

There is a dearth on studies evaluating trends on alcohol consumption in low- and middle-income countries. Therefore, the present study aimed to fill this gap by evaluating the changes in RD during three different moments of the pandemic. The changes were assessed among the Brazilian general population, as well as compared between individuals reporting or not chronic diseases and reporting or not mental health disorders.

## Method

This is a secondary analysis profiting from data obtained in three consecutive, anonymous, web surveys conducted in Brazil: Survey 1 (S1) was conducted from April 20-May 20, 2020^19^; Survey 2 (S2) from August 28-October 9, 2020; and Survey 3 (S3) from January 18-March 6, 2021.^26^ The questionnaire was programmed in SurveyGizmo^®^ and published elsewhere.^27^ Skips were implemented to decrease the time of completion, and the usability of the online version was tested before launching the surveys. All websurveys comprised convenience samples including adults of both sexes, with access to the internet, self-reporting living in Brazil, and who agreed to participate after reading the informed consent. Multiple entries by the same individuals were prevented by asking if the surveys were filled before (S2 and S3). Modified snowball sampling and sponsored social network advertisements were used for recruitment. Due to the lack of parameters to estimate the sample size for S1, a 30 day-period of data collection was pre-specified.

### Outcome

The primary outcome was the change in the prevalence of RD across the three surveys. RD was screened through the Alcohol Use Disorder Identification Test-Concise (AUDIT-C), using a cut-off ≥ 3 (which presents 95% sensitivity and 60% specificity for detecting heavy alcohol use and AUD).^28^

### Main variables of interest

The main variables of interest were self-reported chronic diseases and mental health disorders. Previously diagnosed chronic diseases and mental health disorders were investigated using the question "In the last 12 months, have you been diagnosed by a medical doctor or health professional, or received treatment for (diabetes, heart disease, hypertension, asthma, cirrhosis, kidney disease, cancer, depression, anxiety, schizophrenia, bipolar disorder and eating disorders)?"^29^ Those conditions were aggregated as chronic diseases and mental health disorders, following Salles et al.^24^

### Covariates

Demographic information included sex(male/female), age (dichotomized by the median value, i.e., 41 years), education level (up to high-school/undergraduate/university degree or more), number of people living in the household (1/2-3/4-9), geographic regions (Midwest, North, and Northeast/South and Southeast) – aggregated due to the findings from socioeconomic vulnerability presented by Rocha et al.^30^ and employment (no/yes/unemployed due to COVID-19).

Lifestyle was measured using the Short Multidimensional Inventory Lifestyle Evaluation- Confinement (SMILE-C)^27^ developed and validated to allow a multidimensional measure of lifestyle. It comprises 27 items, evaluating seven domains (Diet and Nutrition, Substance use, Physical activity, Stress management, Restorative sleep, Social support, and Environmental exposures). Answers are provided through a Likert scale and summed up to provide an overall score. The higher the score, the healthier the lifestyle.

### Statistical analysis

We described the absolute and relative frequencies of demographic and clinical characteristics among individuals presenting RD in each survey (S1, S2, and S3). The mean prevalence and their 95%CI of RD in each survey were estimated after bootstrapping the samples; prevalence and their 95%CI of RD were also estimated for each of the variables of interest (i.e, chronic diseases and mental health disorders). Bootstrap samples (B = 2,000) were stratified by sex, age, geographic region, employment, education level, lifestyle, mental health disorders, and chronic diseases totaling 576 strata, equivalent to 1,152,000 stratified bootstrap samples. For each stratum of equal size (n = 100), a sampling weight inversely proportional to its representativeness in the original sample was calculated.

To evaluate the changes in the prevalence of RD, in the first moment, unconditional binomial models were fitted in each bootstrap round in each stratified sample. For each of these models, the pairwise adjusted/marginal odds-ratio (aOR) between the surveys for each outcome were obtained after Tukey's honest significant difference^31^ correction for multiple testing and summarized as mean-marginal aOR bootstrap estimates and their 95%CI. Statistical significance was determined by evaluating using 95%CIs.

All statistical analyses were performed using the software R v.4.2.2 and the libraries "boot," "emmeans" and their dependencies.

### Ethical considerations

The websurveys were approved by the Comissão Nacional de Ética em Pesquisa (CONEP, Brazil – 3.968.686) and the Ethics Committee from Hospital de Clínicas de Porto Alegre (CAAE-31520620.0.1001.5327).

## Results

The original characteristics of the sample with positive screening for by RD are presented in Table 1. In all surveys, RD was more frequent among individuals who did not report chronic diseases. RD was also more frequent among those reporting mental health disorders in S1 and S2, but not S3. RD was also more frequent among men, younger, those with higher educational attainment, and presenting worse lifestyle.

**Table 1:** Characteristics of the sampled participants in S1, S2, and S3 with positive screening for by RD (AUDIT-C score ≥ 3) (Brazil, 2020-2021)

			Survey 1 n = 19,257	Survey 2 n = 1,590	Survey 3 n = 859
Main variables of interest					
	Chronic disease				
		No	6,193 (47.6)	354 (38.5)	225 (39.8)
		Yes	2,534 (41.6)	170 (30.9)	58 (24.9)
	Mental disorder				
		No	5,493 (45.0)	334 (34.9)	191 (35.1)
		Yes	3,057 (46.6)	180 (37.1)	88 (35.5)
Covariates					
	Sex				
		Female	5,873 (44.7)	414 (34.0)	235 (35.0)
		Male	2,914 (47.5)	111 (42.5)	49 (36.8)
Age (years)					
		18-41	5,936 (50.0)	224 (41.7)	153 (38.1)
		41 or more	2,851 (38.6)	301 (32.0)	131 (32.6)
	Region				
		MW/N/NE	1,989 (46.0)	70 (35.0)	20 (26.0)
		S/SE	6,798 (45.5)	455 (35.6)	264 (36.3)
	Employed				
		No	2,982 (40.6)	174 (28.6)	71 (29.0)
		Yes	5,481 (48.4)	341 (40.8)	204 (38.3)
		Unemployed due COVID-19	324 (54.5)	10 (30.3)	9 (33.3)
	Education level				
		Up to high-school	1,996 (43.2)	106 (26.1)	57 (27.5)
		Undergraduate	4,611 (45.4)	271 (36.8)	145 (36.4)
		University degree or more	2,180 (48.6)	148 (44.0)	82 (41.2)
	Household members				
		1	1,241 (49.1)	70 (33.3)	44 (36.1)
		2-3	5,170 (46.3)	337 (36.7)	171 (35.7)
		4-9	2,365 (42.7)	118 (33.8)	68 (33.7)
	Lifestyle				
		Worse	277(60.5)	17 (51.5)	8 (47.1)
		Better	8,510 (45.3)	508 (35.2)	276 (35.1)

Data presented as n (%).

AUDIT-C = Alcohol Use Disorder Identification Test-Concise; COVID-19 = coronavirus disease 2019; MW/N/NE = Midwest/North/Northeast Regions; RD = risky drinking; S = survey; S/SE = South/Southeast Regions.

After bootstrap and adjustment, the overall prevalence of RD was 45.83% (95%CI 45.51-46.15) in S1, 35.28% (95%CI 34.94-35.63) in S2, and 33.69% (95%CI 33.33-34.03) in S3. Additionally, the prevalence of RD was lower among individuals who report chronic diseases (compared to those without it), and the prevalence of RD was higher among those reporting mental health disorders in S1 and S2 (compared to those without it). All these differences are statistically significant, as can be observed by the non-overlap of 95%CIs (Supplementary Table S1).

**Figure 1:**
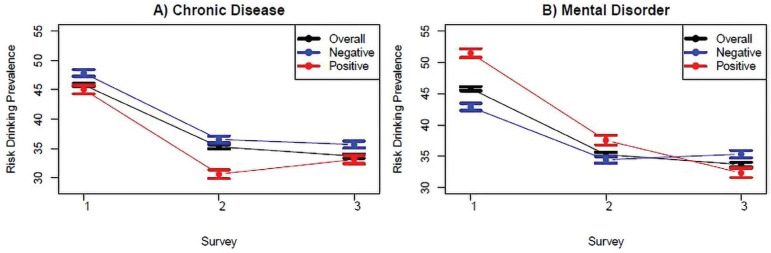
Bootstrapped risk-drinking prevalence estimates* (Alcohol Use Disorder Identification Test-Concise [AUDIT-C] score ≥ 3) in the three surveys, overall and among individuals presenting (or not) chronic diseases, and mental health disorders. Brazil, 2020-2021. * Resampling (B = 2,000) stratified by sex, age, education level, employment, number of people in the household, chronic and mental health diseases, where applicable.

Figure 1 shows the decrease in the prevalence of RD in the three surveys. Among individuals reporting chronic diseases, the prevalence of RD was 45.05% (95%CI 44.31-45.79) in S1 and 33.11% (95%CI 32.38-33.82) in S3, while among those without chronic diseases it was 47.86% (95%CI 47.30-48.42) in S1 and 35.67% (95%CI 35.06-36.26) in S3. Among individuals reporting mental health disorders the prevalence of RD was 51.50% (95%CI 50.78-52.20) in S1 and 32.34% (95%CI 31.62-33.11) in S3, while among those without mental health disorders it was 42.89% (95%CI 42.32-43.47) in S1 and 35.35% (95%CI 34.73-35.96) in S3 (Supplementary Table S1). All the findings were also statistically significant in the adjusted logistic models, as can be observed in Supplementary Table S2, where 95%CIs do not include 1.

## Discussion

In the present longitudinal analysis, we show that there was a decrease in the prevalence of RD in Brazil between April 2020 and March 2021. Additionally, individuals reporting chronic diseases presented a lower prevalence of RD, while those reporting mental health disorders present a higher prevalence (compared with those without chronic and mental health problems, respectively). It is noteworthy that these changes reflect the year after Brazil's first COVID-19 case was diagnosed and not pre-post pandemic changes.

Cross-sectional studies, conducted in the begging of the pandemic, reported mixed results regarding alcohol consumption.^32^ For instance, Moura et al.^13^ observed a 32,5% reduction in alcohol consumption, with a positive association of alcohol consumption with anxiety and depression, both isolated and combined. Malta et al.^14^ also observed a reduction in consumption, but among Brazilian adolescents. In terms of drinking behaviors, Garcia-Cerde et al.^15^ observed a negative association between quarantine and the frequency of heavy episodic drinking. Otherwise, Baptista et al.^33^ and Nin et al.^16^ observed increased alcohol consumption. Here, we employed a longitudinal approach with a validated scale (AUDIT-C) which revealed a reduction in RD along three consecutive waves. Insofar international longitudinal studies have been published, results indicate no changes/decrease in alcohol use and in RD at the general population level.^34^ Although our results do not capture pre-pandemic alcohol use, it is possible that there was an increase in alcohol consumption in the beginning of the pandemic, as reported by Nin et al.^16^ possibly reflecting as a mechanism cope with high levels of stress/ anxiety,^35^ followed by regression towards pre-pandemic mean.^36^ Additionally, there were changes in on/off-premises alcohol consumption that may have influenced drinking patterns. For instance, in February-March 2021, Brazil was experiencing the second COVID-19 wave, and although the levels of anxiety/stress could be lower than in the 2020, there was a second increase in proportion of people staying at home (see the changes in the Brazilian Home Stay Index at https://bigdata-covid19.icict.fiocruz.br/). It is possible, as suggested by our previous findings,^18^ that the decreased opportunities of social drinking may have decreased RD.^37^ Considering the multiple macro- and individual- level factors that influence drinking behavior, future studies to disentangle these effects are necessary.

The decreasing prevalence of RD in the 1st year of the pandemic, however, is not completely reassuring. Even before the pandemic, Case and Deaton^38^ pointed to the increase in alcohol related mortality among white, US men. Notably, the increase disproportionally affected those with lower educational attainment, and could be related to the increase on social inequalities and poverty. In Brazil, the economic consequences of the pandemic worsened the scenario imposed by austerity measures implemented since 2015.^39^ These measures affected social security and the Brazilian public unified health system, and investigating how those macro-level determinants of health will impact RD, and its consequences, is of utmost importance for planning public health policies in the next years.

The higher prevalence of RD among people with mental health disorders corroborates previous reports pointing to a higher probability of AUD in people with psychiatric comorbidities.^40^ The reasons behind this frequent comorbidity are still debated, be it because of a direct or indirect causal effect of AUD on other psychiatric disorders or the other way around, because of shared environmental and genetic causes or shared psychopathological mechanisms.^41,42^

Individuals with chronic disease, in turn, presented a lower prevalence of RD. This result is in contrast with data from the pre-pandemic period, as reported by Pham et al.,^43^ who found similar drinking patterns in people with chronic disease and the general population. Manthey et al.^25^ reported a reduction of alcohol use in Colombia's and Mexico's primary care patients, suggesting the lack of social gatherings, a restrictive set of alcohol control policies, and economic losses as possible explanations for their findings. Even so, alcohol consumption still negatively impacts one's treatment, either because there is a negative association between medication adherence and alcohol use^44^ or because the substance itself can worsen the clinical condition.^45^ Thus, monitoring consumption among clinical populations continues to be important for providing AUD treatment and for planning health services.

The study is not free of limitations. First, the sample was non-probabilistic, as many other websurveys,^46^ and results may not be generalized to the entire population. All non-probability/convenience samples are prone to selection bias and the effects brough by this bias are uncertain. For instance, it is possible that individuals presenting mental health problems were more likely to answer, which would increase RD prevalence. On the other side, it is also possible that individuals presenting physical problems were more likely to answer, decreasing the RD prevalence. The only way to control selection bias is to conduct a probability study, but this still represents a challenge when using web surveys – and during the pandemic it even more difficult, considering the time and financial resources needed for conducting those studies.^46,47^ Second, this is a longitudinal study, but the samples do not comprise the same individuals. Although cohorts are also prone to selection bias, and attrition, the evaluation of changes on RD at the individual level should be confirmed using a cohort designs investigation. Third, women, who usually drink less than men,^3^ were overrepresented, but we were able to control the outcomes by gender. Fourth, individuals presenting lower socio-economic resources – and more prone to the pandemic's economic consequences-may be underrepresented. The effect of this selection bias will have to be examined profiting from additional studies employing different sampling methods (respondent driving sampling, for example). Finally, social desirability bias regarding alcohol use may not be excluded but considering the normalization of alcohol drinking behavior,^3^ it is unlikely that answering the AUDIT-C would make individuals embarrassed.

Despite the limitations, this is the first study to bring information with longitudinal data from a continental middle-income country in Latin-American after the COVID-19 outbreak. Although there was decrease on RD prevalence, the economic consequences of pandemic and the dismantlement of the unified health system, make the monitoring of alcohol use trends crucial for public health policies.
