# Supplementary material for: Changes on risky drinking after the COVID-19 outbreak in Brazil: results from three consecutive web surveys
Source: Trends Psychiatry Psychother. 2025 May 26;47:e20230686. doi: 10.47626/2237-6089-2023-0686 (PMC12916106; doi:10.47626/2237-6089-2023-0686)
Supplement: Supplementary file 1 [file 2238-0019-trends-47-e20230686-Suppl01.pdf]

http://doi.org/10.47626/2237-6089-2023-0686

Supplementary Table S1 - Adjusted prevalence and 95%CI of RD along the surveys, overall and by chronic and mental health diseases (Brazil 2020-2021)

Survey	Category		Prevalence (95%CI)
1	Overall		45.83 (45.51-46.15)
2	Overall		35.28 (34.94-35.63)
3	Overall		33.69 (33.33-34.03)
1	Chronic disease	Negative	47.86 (47.30-48.42)
2	Chronic disease	Negative	36.53 (35.92-37.12)
3	Chronic disease	Negative	35.67 (35.06-36.26)
1	Chronic disease	Positive	45.05 (44.31-45.79)
2	Chronic disease	Positive	30.62 (29.85-31.32)
3	Chronic disease	Positive	33.11 (32.38-33.82)
1	Mental disorder	Negative	42.89 (42.32-43.47)
2	Mental disorder	Negative	34.46 (33.91-35.03)
3	Mental disorder	Negative	35.35 (34.73-35.96)
1	Mental disorder	Positive	51.50 (50.78-52.20)
2	Mental disorder	Positive	37.56 (36.81-38.36)
3	Mental disorder	Positive	32.34 (31.62-33.11)

95%CI = 95% confidence interval; RD = risky drinking.
Resampling (B = 2,000) stratified by sex, age, education, employment, number of people in the household, chronic and mental health diseases, where applicable.

Supplementary Table S2 - Bootstrapped estimates (aOR) for RD along with the surveys, by chronic and mental health diseases (Brazil, 2020-2021)

	Survey	aOR (95%CI)
Chronic disease		
No	2/1	0.63 (0.61-0.65)
No	3/1	0.60 (0.58-0.63)
No	3/2	0.96 (0.93-1.00)
Yes	2/1	0.54 (0.51-0.56)
Yes	3/1	0.60 (0.58-0.63)
Yes	3/2	1.12 (1.07-1.18)
Mental disorder		
No	2/1	0.70 (0.68-0.72)
No	3/1	0.73 (0.70-0.76)
No	3/2	1.04 (1.01-1.08)
Yes	2/1	0.57 (0.54-0.59)
Yes	3/1	0.45 (0.43-0.47)
Yes	3/2	0.79 (0.76-0.83)

95%CI = 95% confidence interval; aOR = adjusted/marginal odds-ratio; RD = risky drinking.
Resampling (B = 2,000) stratified by sex, age, education, employment, number of people in the household, chronic and mental health diseases, where applicable.
